# Sustainable Geothermal Silica Quantum Dots Synthesized via Acid–Base Hydrothermal Method for Selective Melanoma Theranostics and Bioimaging

**DOI:** 10.1021/acsomega.5c06202

**Published:** 2025-12-02

**Authors:** Novi Irmania, Solihin Solihin, A’liyatur Rosyidah, Dito Anurogo, Farizal Hakiki

**Affiliations:** † 599846 National Research and Innovation Agency (BRIN), Research Center for Geological Resources, Bandung 40135, Indonesia; ‡ National Research and Innovation Agency (BRIN), Research Center for Vaccine and Drug, Bogor 16911, Indonesia; § 482621 Universitas Muhammadiyah Makassar, Faculty of Medicine and Health Sciences, Makassar 90221, Indonesia; ∥ 34914 National Yang Ming Chiao Tung University, Civil Engineering Department, Hsinchu 300, Taiwan; ⊥ National Yang Ming Chiao Tung University, Disaster Prevention and Water Environment Research Center, Hsinchu 300, Taiwan

## Abstract

This study reports the synthesis and characterization of silica quantum dots (silica QDs) derived from geothermal-waste silica sourced from the Dieng Geothermal Field, Central Java Province, Indonesia. It supports green chemistry by converting abundant waste into valuable nanomaterials, contributing to sustainable energy and material recovery. Characterization using field emission scanning electron microscopy (FESEM), high-resolution transmission electron microscopy (HRTEM), and dynamic light scattering (DLS) confirms the formation of spherical silica QDs with a uniform size distribution between 2–5 nm, averaging 3 nm. A zeta potential of −28 mV indicates strong colloidal stability in both suspension and biological media. Silica QDs exhibit excitation-dependent photoluminescence with a 20% quantum yield, making them suitable for applications such as bioimaging and photoresponsive drug delivery. In vitro results show selective cytotoxicity against B16F0 melanoma cells while sparing NIH3T3 normal fibroblasts, indicating biocompatibility and potential for targeted therapy. These findings reveal the dual role of silica QDs as diagnostic and therapeutic tools. This work reinforces the link between sustainable nanomaterial synthesis and biomedical innovation, illustrating how waste-to-resource strategies can drive advances in nanomedicine.

## Graphical abstract

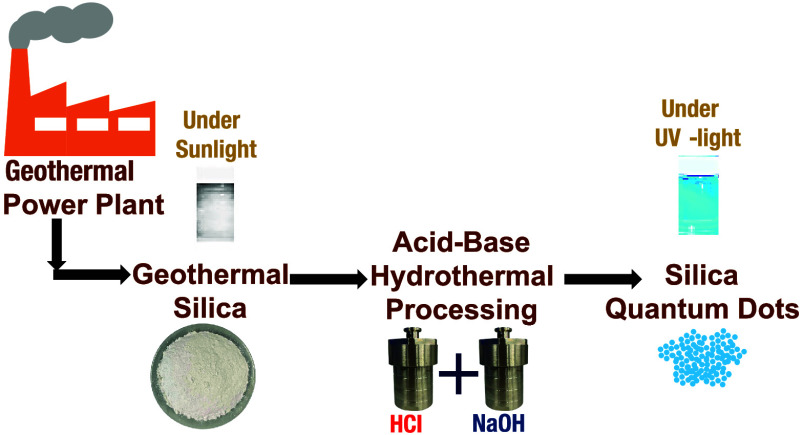

## 1 Introduction

Nanotechnology has transformed materials science by allowing the development of sophisticated nanostructures with remarkable physical, chemical, and biological capabilities.^1^ Quantum dots (QDs) are notable for their distinct photoluminescence, high quantum yield, and tunable optical properties, making them useful in a wide range of applications, from optoelectronics to healthcare.^2,3^ Moreover, QDs have excellent biocompatibility, making them ideal for biomedical applications, particularly customized medicine.^4^ For example, the functionalization of QDs with breast cancer antibodies such as MUC-1 has shown great promise for developing tailored diagnostics and therapeutic techniques.^5,6^ By embedding QDs in biocompatible matrices, their stability under physiological settings is improved, expanding their real-world biomedical applications.^7^

Silica quantum dots (silica QDs), in particular, have received a lot of interest due to their high photoluminescence efficiency, biocompatibility, and stability, making them excellent for bioimaging, diagnostics, and targeted drug delivery.^8,9^ The silica shell improves optical performance while simultaneously reducing cytotoxicity, assuring compatibility with biological systems.^10^ However, common silica QDs synthesis usually uses expensive and nonsustainable chemical precursors, such as tetraethyl orthosilicate (TEOS), restricting their scalability and widespread applicability in clinical and industrial settings. Recent research highlights the necessity for sustainable and cost-effective means of producing QDs, spurring investigations into natural sources and novel synthesis processes. There is growing interest in developing sustainable and green synthesis approaches that utilize naturally abundant and low-cost silica sources.^11^

Silica, primarily composed of silicon dioxide (SiO_2_), is one of the Earth’s most abundant compounds, naturally occurring in forms such as quartz and silicate minerals.^12^ Noncrystalline silica is created through the erosion and decomposition of silica-based rocks, followed by the dissolution and precipitation of SiO_4_^2–^ ions.^12,13^ Amorphous silica, the most reactive form of silica, can be found in the suspension of geothermal fluid beneath geothermal areas like Dieng Mountain in Central Java, Indonesia. Historically considered waste, this geothermal silica is now seen as a potential resource, contributing to sustainable practices. The polymerization of monomeric silica from geothermal wastewater is an example of a circular economy strategy, transforming industrial waste into valuable resources while reducing the environmental impact.^14,15^ The recycling of silica from sources such as geothermal energy production is gaining prominence as a sustainable practice.^16^ Unlike traditional silica waste sources like rice husk ash, fly ash, or diatomaceous earth, geothermal silica is naturally amorphous and contains over 98% pure SiO_2_, reducing the need for pretreatment.^5,17^ Geothermal silica occurs naturally in colloidal form and is rich in surface silanol (Si–OH) groups, which increases its reactivity under hydrothermal conditions and accelerates conversion into silica QDs. Its strong reactivity, low impurity levels, and ease of dispersion make it ideal for direct conversion to high-quality silica QDs via our acid–base hydrothermal method.^14^

Several waste-derived silica sources have been investigated as precursors for the synthesis of silica QDs using a variety of methods, including sol–gel, microwave-assisted synthesis, pyrolysis, and alkaline extraction.^18–22^ However, each of these approaches has significant drawbacks when compared to the acid–base hydrothermal method. The sol–gel technique, while adaptable, frequently necessitates multistep processing and produces broad particle size ranges with inferior crystallinity unless post-treatment is used.^23,24^ Microwave-assisted synthesis provides fast reaction kinetics, but it frequently requires organic solvents or surfactants, suffers from uneven heating, and is difficult to scale up effectively.^20,21^ Pyrolysis procedures need high temperatures (>700 °C), which are energy-intensive and can produce carbon impurities that affect the optical clarity and surface purity of QDs.^25^ Alkaline extraction methods, while effective for silica recovery, generate significant chemical waste and produce amorphous silica with less control over the shape and surface functionality.^26,27^

The synthesis of silica QDs from geothermal silica via an acid–base hydrothermal method presents significant advantages over other environmentally friendly approaches. This approach allows for gentle reaction conditions in aqueous media at moderate temperatures, without the need for high-temperature calcination or harmful reducing chemicals.^28^ This not only reduces energy consumption but also eliminates the use of organic solvents and high-temperature calcination steps.^13^ Additionally, the method is highly scalable due to the availability of geothermal silica in bulk quantities and its amenability to direct use without the need for pretreatment.^29^ It also enables great scalability and reproducibility, allowing for precise control over particle morphology and photoluminescent characteristics, which are critical for large-scale production and biomedical applications.^30,31^ These benefits highlight the originality and sustainability of this strategy for converting geothermal waste into high-performance, biocompatible nanomaterials. Overall, this green synthesis strategy offers a reproducible, scalable, and functionally superior route for producing high-quality silica QDs tailored for advanced nanobiotechnology applications.

Therefore, this study aims to develop an environmentally friendly approach to synthesize silica quantum dots (QDs) using mineral-based silica precursors derived from geothermal residue at Dieng Geothermal Power Plant, Central Java, Indonesia. The acid–base hydrothermal approach is an efficient and scalable method to produce QDs.^32^ This technique allows for exact control over particle size, shape, and optical properties, resulting in high-quality nanomaterials.^33–35^ Comprehensive physicochemical properties characterization of the synthesized silica QDs in this study includes size, shape, and optical features. The latter property is essential for the biomedical application. Finally, we conduct cytotoxicity assessments to evaluate the potential of silica QDs for bioimaging and theragnostic therapy.

## 2 Materials and Methods

### 2.1 Materials

We initially extracted silica mineral from a geothermal fluid yielded by a geothermal power plant site in Dieng Mountain, Wonosobo, Central Java, Indonesia. The silica was rinsed with double-distilled water to eliminate salt before being dried for 24 h. The chemicals used in this experiment were hydrochloric acid (HCl, 36%, *d* = 1.18 g/L) and sodium hydroxide (NaOH) purchased from Merck. We used stainless steel autoclaves, ultrasonic baths, centrifuges, and hydrothermal Teflon to synthesize and purify composite silica QDs. All of the compounds were analytical grade and usable without further purification.

### 2.2 Synthesis

First, 2 g of silica was added to 50 mL of HCl (2 M) and rapidly agitated for 2 h. The mixture was then moved to a Teflon-lined stainless-steel autoclave, where the hydrothermal reaction took place for 18 h at 200 °C. The resulting solution was cooled to ambient temperature. The cooled solution was centrifuged with deionized water three times at 6000 rpm for 15 min to eliminate any impurities before drying in the oven for 3 h. After drying, the powder was added to 50 mL of NaOH (0.1 M) and agitated for 2 h until well combined. The well-combined mixture was transferred to another Teflon reactor and heated at 200 °C for 18 h. The solution was cooled to room temperature and filtered through a 200 nm syringe filter. Finally, we achieved a silica QDs suspension. The synthesis procedure was developed based on systematic preliminary trials that explored variations in reaction temperature and acid–base treatment duration to maximize yield and photoluminescence intensity.

### 2.3 Characterizations

The chemical composition of the silica quantum dots was determined using X-ray fluorescence (XRF) equipment (S2 Puma Bruker). The minerals in the silica powder were analyzed using X-ray diffraction (XRD) equipment (Malvern Panalytical-Benchtop XRD Aeris 600 W) using CuK-alpha radiation at a voltage and current of 40 kV and 30 mA. The sampling pitch and scanning speed were 0.02° and 2.4°/min. A field emission scanning electron microscope (FE-SEM, brand: Apreo 2 S) equipped with energy-dispersive X-ray spectroscopy (EDS) was used to reveal the morphology of the powder and the distribution of atoms within it. The morphology, size, and crystal structure of Silica QDs were examined using high-resolution transmission electron microscopy (HRTEM, brand: TEM Talos F200X) with an energy-dispersive X-ray spectroscope (EDS) operating at 200 kV. The sample’s absorbance-wavenumber profile was determined using Fourier transform infrared spectroscopy (FTIR, brand: Thermo Scientific Clever iTX ATR Accessory for the Nicolet) in the 500–4000 cm^–1^ range. We used a dynamic light scattering apparatus (DLS, brand: Malvern Zetasizer Nano-ZS) to determine the size of silica QDs in dispersion and the dispersive properties after being dissolved in double-distilled water. UV–vis absorption spectra at room temperature were obtained with a JASCO V630 spectrometer. Photoluminescence (PL) spectra were obtained at room temperature using a Fluorolog-3 spectrophotometer fitted with a 450 W xenon lamp.

### 2.4 Cell Culture Maintenance

B16F0 melanoma and NIH-3T3 fibroblast cell lines were purchased from ECACC and grown in Dulbecco’s Modified Eagle’s Medium (DMEM, Sigma-Aldrich), 1% antibiotic/antimycotic, and 10% fetal bovine serum (FBS, Sigma-Aldrich). Both cell lines were incubated at 37 °C in a humidified incubator with 5% CO_2_ supplementation. The cells were subcultured every 3 days. The cells were grown in 96-well plates before cytotoxicity testing.

### 2.5 Cytotoxicity Assay

To assess the effect of silica QDs on cell viability, the MTT assay was used. Cells were seeded onto 96-well plates at a concentration of approximately 1.0 × 10^4^/well in 200 μL of culture media and incubated at 37 °C in a humidified incubator with 5% CO_2_ for 24 h. The medium in the wells was then replaced with fresh serum-free medium containing silica QDs at various concentrations (15.6, 31.2, 62.5, 125, 250, 500, and 1000 ppm). The medium was removed after 24 h of incubation at 37 °C with 5% CO_2_, and MTT (10 μL, 5 mg/mL in PBS solution) was added to the treated cells and incubated at 37 °C for another 4 h under dark conditions. After incubation, 100 μL of DMSO was added to the MTT-containing solution to dissolve the precipitated formazan crystals. The control group cells were given PBS without silica QDs, and viability was defined as 100%. The cell viability was assessed by measuring the cell absorbance at 570 nm with a UV–vis spectrophotometer (Multiskan, Thermo Fisher Scientific). Every condition was repeated three times. Cell viability (%) was calculated as the absorbance ratio of the cells after various treatments to the control cells without treatment.

## 3 Results and Discussion

### 3.1 Chemical Compositions

Silica quantum dots (QDs) were synthesized via a hydrothermal method, as depicted in Figure 1. The synthesis utilized geothermal silica extracted from scale deposits in the Dieng geothermal field, Wonosobo, Central Java, Indonesia. This geothermal byproduct is rich in reactive, amorphous silica and contains relatively low levels of contaminants, making it a promising sustainable precursor. To improve the purity and overall quality of the resulting silica QDs, the process incorporated hydrochloric acid (HCl) treatment. This acid leaching step effectively removed metallic impurities, particularly iron (Fe) and manganese (Mn), which are known to adversely affect key physical properties of silica materials, including surface area, porosity, and particle size.^36^ The elimination of these contaminants is essential, as their presence can significantly compromise the performance of silica-based nanomaterials across various applications. The acid-treated material then underwent hydrothermal synthesis to produce high-purity silica QDs with favorable optical and structural characteristics, rendering them suitable for biomedical applications.^37^

**Figure 1:**
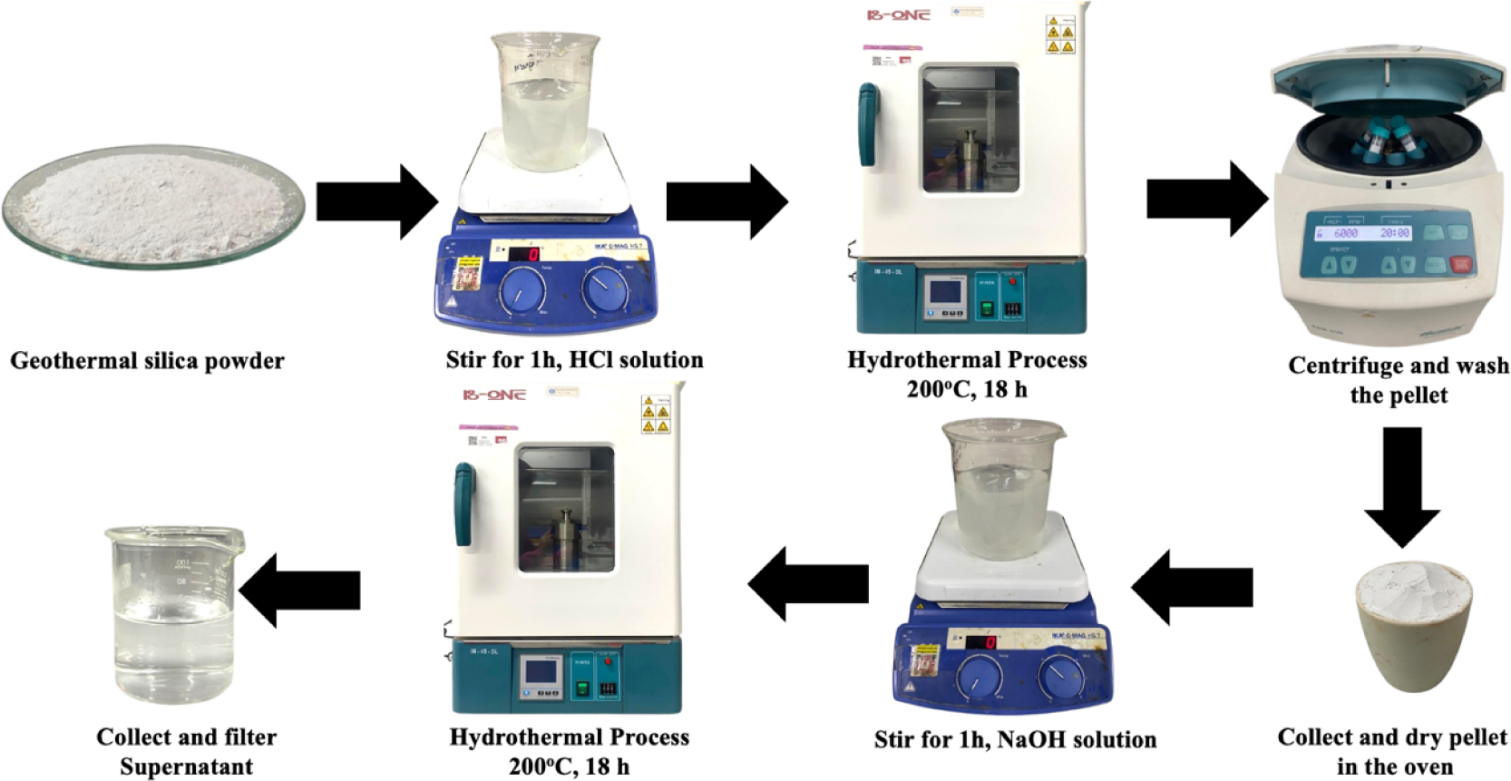
Schematic of synthesis workflow: from geothermal silica to silica QDs.

In the synthesis of silica QDs, geothermal silica served as the silicon source, while HCl acted as the acid etchant and NaOH as the alkaline etchant. The initial step involved hydrothermal treatment under acidic conditions using HCl, which effectively dissolved impurities and enhanced the purity of the silica.^38^ Since geothermal silica contains a significant amount of silicon and oxygen in its structure, hydrochloric acid was particularly effective in leaching metal ions and removing impurities while preserving the silica framework. A 2 M HCl concentration was selected to ensure sufficient protonation of the surface silanol groups in the geothermal silica. HCl facilitates the breakdown of metal oxide impurities and promotes the release of orthosilic acid Si­(OH)_4_ from the amorphous silica network, a crucial precursor for the nucleation of silica QDs.^39^ Prior literature also supports this concentration range as effective for achieving high reactivity while avoiding overetching that could result in particle aggregation or structural collapse.^29,36^

Following the acid treatment, the silica underwent a secondary hydrothermal step using 0.1 M NaOH. This lower-concentration alkaline treatment was intentionally chosen to initiate a controlled base-catalyzed condensation of Si­(OH)_4_, leading to the formation of silica QDs with a narrow size distribution. The use of a higher concentration could lead to excessive agglomeration or uncontrolled growth. At 0.1 M, NaOH provides enough hydroxide ions to selectively cleave Si–O–Si bonds into terminal silanol groups (Si–OH), via a nucleophilic attack mechanism without damaging the integrity of forming nanoparticles. This approach mirrors the principle of a “scissoring effect”, where hydroxide ions act on bridging oxygen atoms between two silicon centers, effectively depolymerizing the silica network in a controlled manner. This depolymerization forms smaller silicate species (e.g., Si­(OH)_4_), which subsequently recondense into uniform silica QDs under hydrothermal conditions.^40^ NaOH eliminates any leftover metal ions and promotes the fragmentation of siloxane linkages, enabling the formation of uniform silica QDs.^41^

The hydrothermal process was conducted at 200 °C for 18 h, based on prior studies demonstrating this temperature and time duration as optimal for promoting homogeneous nucleation and controlled growth of silica QDs without the need for calcination or surfactants. The hydrothermal temperature and duration were selected to facilitate the crystallization and self-assembly of silica QDs under saturated vapor pressure. At 200 °C, silanol condensation and particle nucleation occur optimally within 18 h, producing monodispersed QDs. Shorter durations (e.g., 6–12 h) yielded lower PL intensity, while longer durations caused size broadening. This temperature inhibits the formation of large aggregates and favors the development of photoluminescent silica QDs in the desired nanoscale range.^30^ After hydrothermal synthesis, repeated deionized water washing steps were applied postsynthesis to remove residual salts, particularly NaCl, until the supernatant reached near-neutral pH. This acid–base hydrothermal method offers a low-cost, scalable, and environmentally benign approach aligned with sustainable nanotechnology principles by using abundant natural silica sources and eliminating hazardous reagents.^11^ The extraction and synthesis of silica QDs from silica geothermal demonstrate the method’s potential for mass manufacture of high-quality nanomaterials for advanced applications.

The X-ray diffraction (XRD) investigation of silica scaling from geothermal sites and silica QDs gives important information on their crystalline structure and phase transitions. The rough broad humped peak at 2θ of 15–30° for the original geothermal silica (Figure 2a) implies a primarily amorphous structure, as seen in natural silica deposits.^42,43^ In contrast, silica QDs show a smoother, broad humped peak at the same range of 2θ (Figure 2b), indicating that, despite the structure still being amorphous, the solvothermal manufacturing procedure has improved structural ordering, which is among the unique qualities needed in several applications, such as drug delivery and bioimaging.^33^ The three highest peaks at 31.76°, 45.49°, and 56.53°, corresponding to sodium chloride (NaCl, ICDD no. 96-900-3310), indicate that NaCl crystals were formed during the synthesis process.

**Figure 2:**
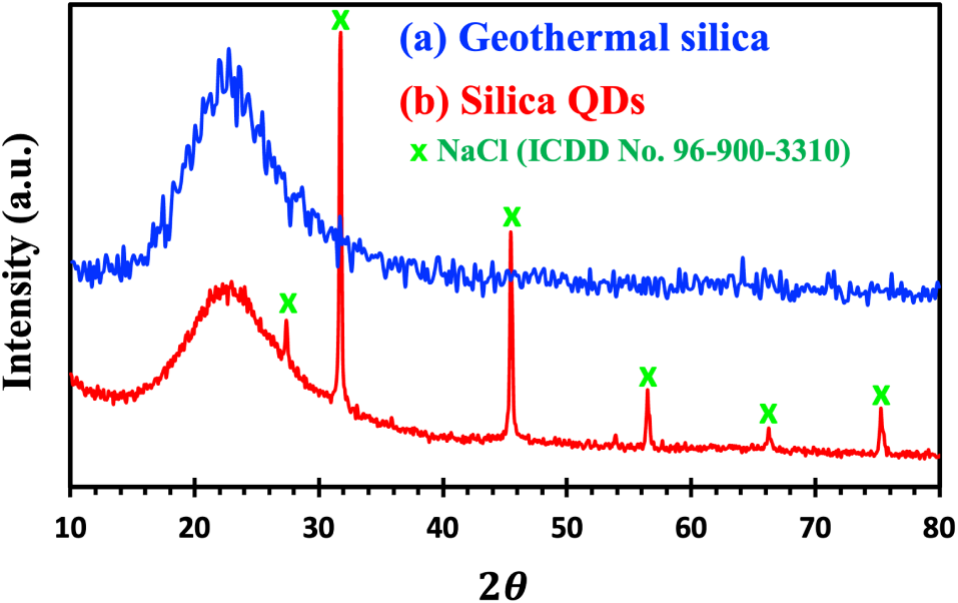
X-ray diffraction patterns of (a) geothermal silica mineral and (b) silica QDs.

We assess the chemical composition of silica QDs, except for H_2_O concentration, with the use of X-ray fluorescence (XRF). Table 1 lists possible minerals found in the silica QDs powder and concludes a high purity level in a SiO_2_ concentration of 92.6 wt %. Impurities such as MgO, Al_2_O_3_, and P_2_O_5_ are typical minerals that are associated with a natural geothermal environment.^44^ Notably, NaCl is the main contaminant, accounting for 4.1 wt %, and the XRD analysis confirms these peaks in the silica QDs powder. NaCl crystals in QDs have the potential to influence the final composition and overall properties of silica QDs, including their stability and interaction with biological systems.^45,46^ This leftover NaCl originates during the neutralization stage of the acid–base hydrothermal synthesis, in which NaOH was used as the alkaline agent. Incomplete washing or crystallization during the drying process may have resulted in the retention of these salts on the QD surfaces. Nonetheless, in this investigation, the optical behavior and morphological features of silica QDs, such as high photoluminescence intensity, spherical shape, and limited size distribution, appear to be uninfluenced by these salt residues.

**Table 1:** Chemical Composition of Silica QDs

**Element**	**SiO**_**2**_	**NaCl**	**MgO**	**Al**_**2**_ **O**_**3**_	**P**_**2**_ **O**_**5**_
**Conc. (wt%)**	92.6	4.1	1.2	1.1	1

The FTIR spectra of the synthesized silica quantum dots (QDs), as depicted in Figure 3, exhibit several characteristic absorption bands. A prominent peak at approximately 667 cm^–1^ corresponds to the O–Si–O bending vibrations, while the strong absorption band around 1080–1200 cm^–1^ is attributed to asymmetric stretching of Si–O–Si bonds, indicating the formation of a silica network structure.^47–50^ Additionally, a shoulder peak near 960 cm^–1^ is associated with the Si–OH stretching vibration, suggesting partial hydroxylation of the surface, which is typical in amorphous silica nanostructures.^49^ The absorption band observed at 1638 cm^–1^ arises from the bending vibration of H–O–H, representing adsorbed water molecules on the QDs’ surface. Moreover, the broad absorption band in the region of 3329–3404 cm^–1^ is characteristic of O–H stretching vibrations, which originate from surface hydroxyl groups and physisorbed water. These hydroxyl functionalities enhance the hydrophilicity and colloidal stability of the QDs in aqueous environments.^48,50^ The presence of surface −OH groups also provide anchoring sites for further surface modification, making the silica QDs suitable for biomedical and environmental applications.

**Figure 3:**
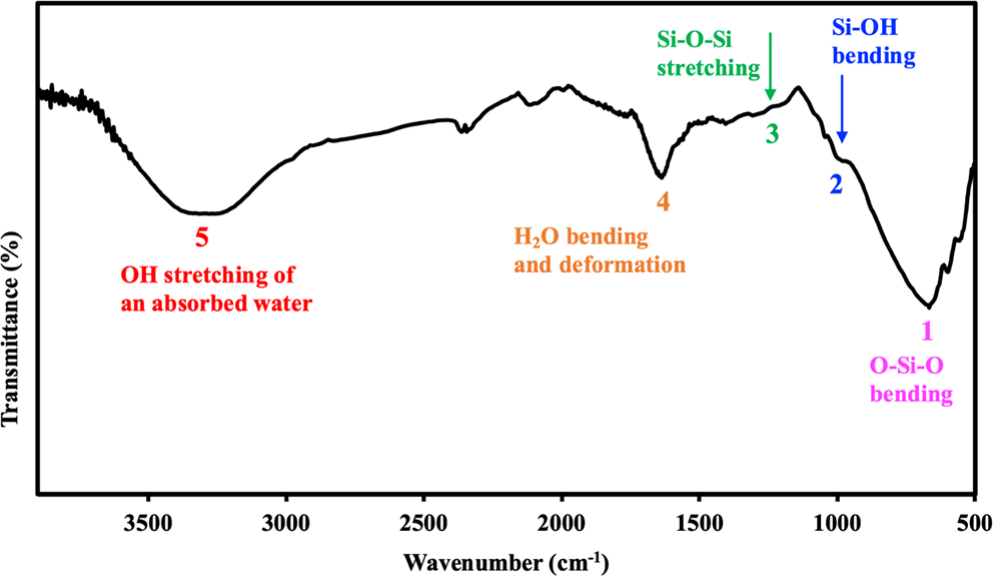
FTIR spectrum of silica QDs.

Furthermore, the Energy-dispersive X-ray (EDX) examination confirms the composition: an atomic percentage of 32.7% Si, 66.2% O, 0.9% Na, and 0.2% Al (Table 2). The presence of carbon originates from the conductive coating.

**Table 2:** Energy Dispersive X-Ray Spectroscopy (EDX) Analysis of Silica QDs

**Element**	**Atomic %**	**Weight %**
Si	32.7	45.8
O	66.2	52.8
Na	0.9	1.2
Al	0.2	0.1

### 3.2 Morphology and Size

Figure 4a,b displays silica QDs morphology measured with field-emission scanning electron microscopy (FESEM) at 150,000× and 250,000× magnification. The FESEM images indicate that the silica QDs possess homogeneous particle sizes and spherical shapes. Further investigation of silica QDs chemistry via energy-dispersive X-ray spectroscopy (EDX) mapping shows that the presence of higher intensity for Si and O at sites confirms the major elements (Figure 4c,d). Meanwhile, lower intensity for Na and Al as minor and trace elements (Figure 4e,f). The presence of carbon originates from the conductive coating.

**Figure 4:**
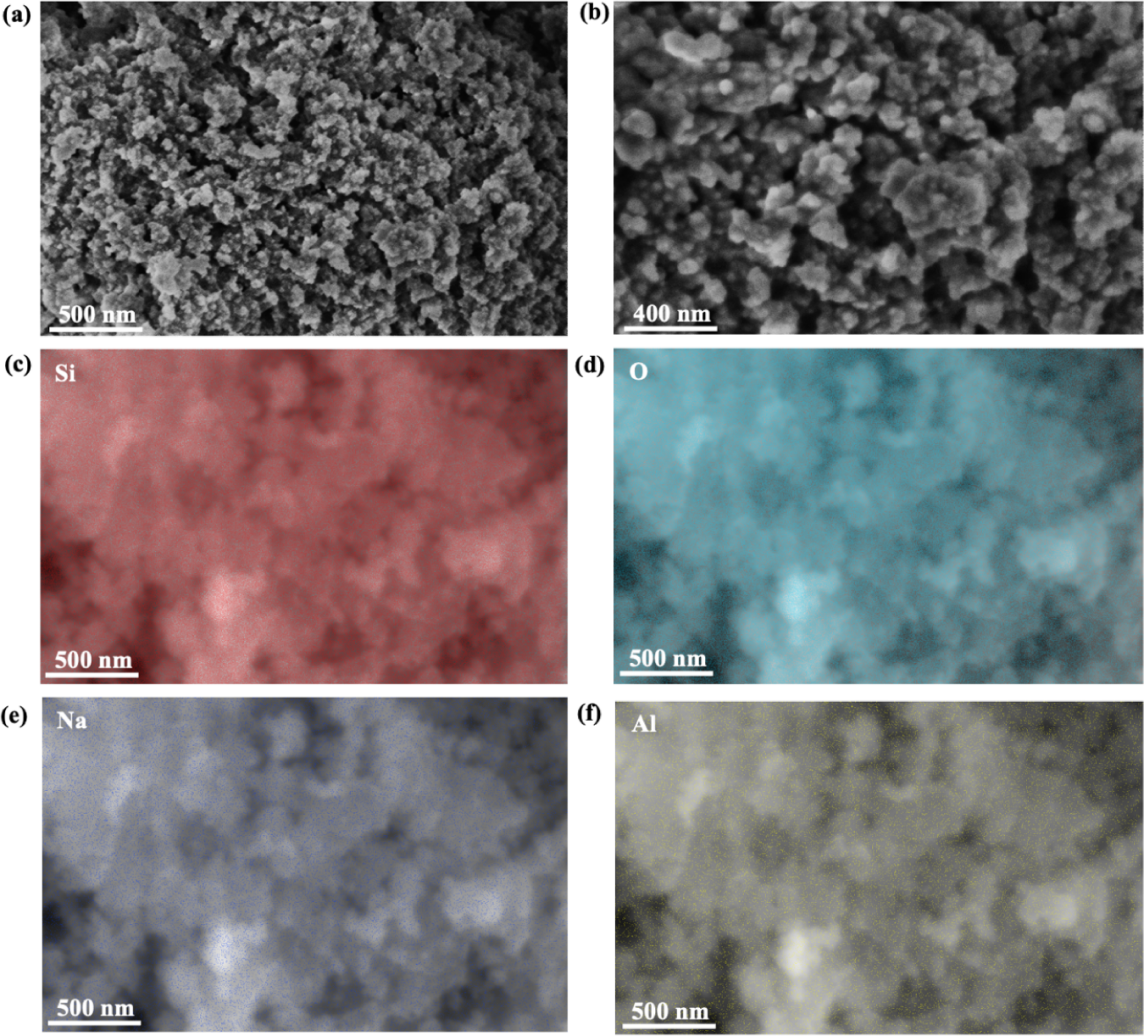
FE-SEM micrographs of silica QDs obtained from (a) 150,000× and (b) 250,000×; FESEM-EDX maps of (c) Si element, (d) O element, (e) Na element, and (f) Al element.

High-resolution transmission electron microscopy (HRTEM) examination of silica QDs yields useful information about their morphological and structural properties. The silica QDs are spheroidal particles that are stable and uniformly dispersed in aqueous solutions (Figure 5a). The presence of distinct fringes at higher magnification indicates well-defined lattice structures within the silica QDs with measured *d*-spacing values of 0.215 nm (inset in Figure 5a). This *d*-spacing of the lattice structure indicates the particle size whose distribution is within a range of 2 to 4.8 nm and averaged at 3.1 nm (Figure 5b). This restricted size distribution is especially useful in biomedical applications, as smaller, uniformly sized silica QDs are associated with increased cellular absorption and lower toxicity.^51,52^

**Figure 5:**
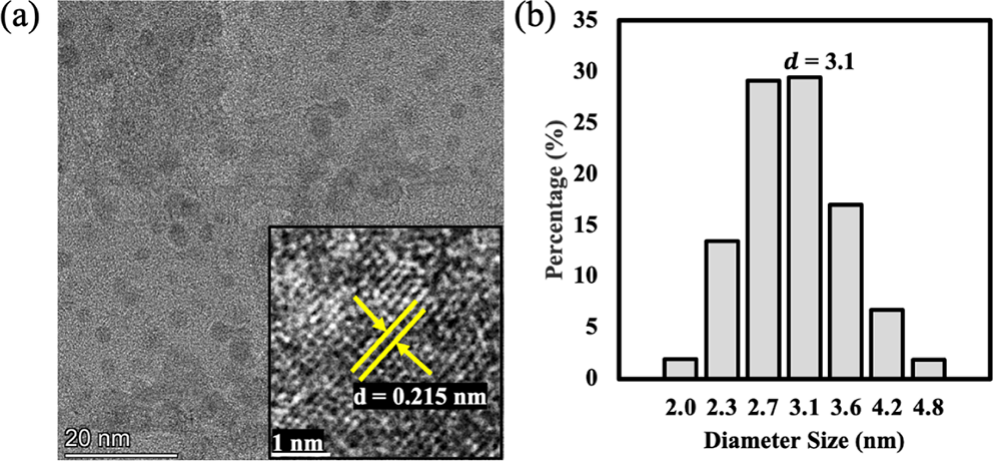
Morphology of silica QDs: (a) HRTEM. Inset: high magnification image and (b) particle size distribution.

Dynamic light scattering (DLS) measurements signify that the silica QDs have an average size of 3.5 nm and a size distribution of 2 to 6 nm (Figure 6a). The single peak and narrow band denote that the particles have a homogeneous size distribution and are monodisperse. This size distribution is critical for guaranteeing uniform behavior in biological applications, as smaller, monodispersed nanoparticles often have higher cellular absorption and lower toxicity, making them better suited for biomedical applications.^53^

**Figure 6:**
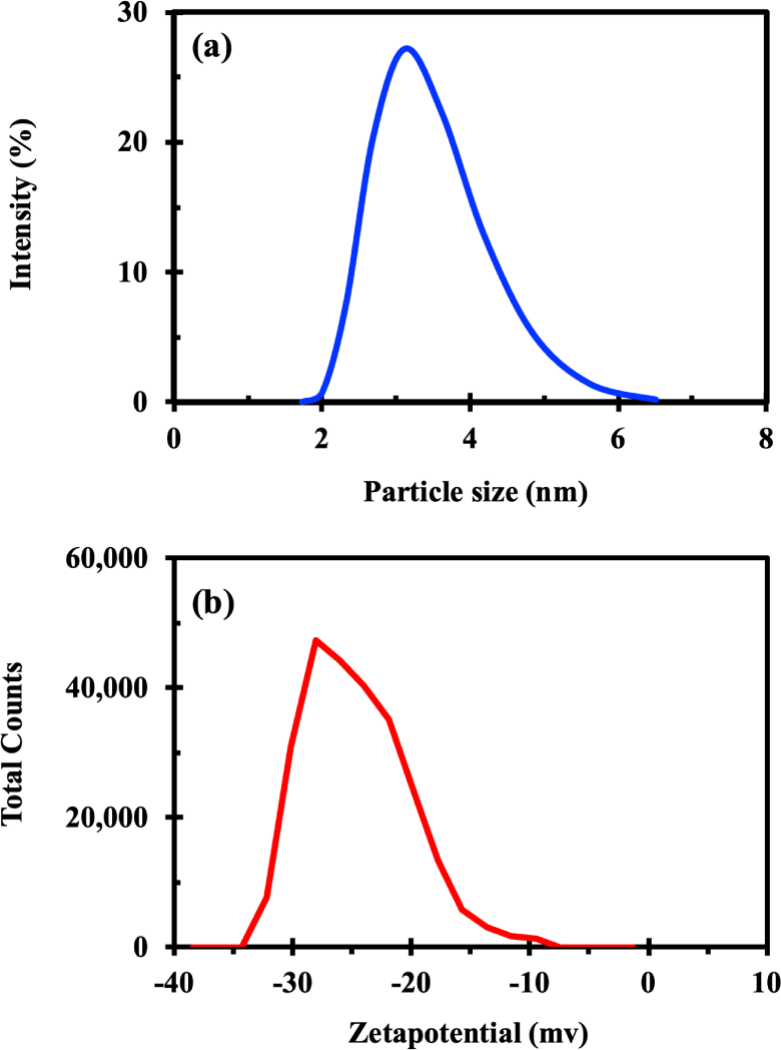
Dynamic light scattering measurements: (a) sizes of silica QDs obtained from particle size analyzer (PSA) and (b) zeta potential graph.

### 3.3 Stability

Figure 6b outlines that the zeta potential of silica QDs is around −28 mV, showing substantial electrostatic repulsion among the nanoparticles that enhances colloidal stability. Therefore, silica nanoparticles are well-known to acquire a negative surface charge when dispersed in aqueous media at pH levels above their point of zero charge. This negative charge primarily arises from the deprotonation of surface silanol groups (≡Si–OH), which lose protons (H^+^) to form negatively charged ≡Si–O^–^ species when the solution pH exceeds the point of zero charge.^54–57^ The point of zero charge of silica typically lies between pH 2 and 4, meaning that at neutral or slightly alkaline conditions (as in our experiments, pH ≈ 7.5), the surface becomes increasingly anionic due to extensive deprotonation.^57–59^ The extent of this deprotonation increases with pH, particularly above the p*K*_a_ range of 2–3 associated with these functional groups, resulting in a stable negative charge even in neutral to mildly alkaline conditions.^60^ Therefore, negative-to-negative Coulombic forces-induced repulsions provide a stable colloidal suspension.^61^

Such surface chemistry is characteristic of colloidal silica systems and results in high negative zeta potential values, often reported between −30 to −50 mV at pH 6–10, reflecting a strongly anionic surface that enhances colloidal stability and may influence biological interactions.^62,63^ In this study, the zeta potential of the silica QDs (−28 mV) aligns well with these values, confirming stable dispersion driven by strong negative-to-negative electrostatic repulsion. When the zeta potential exceeds ± 20 mV, particles have a large surface charge and cause a considerable electrostatic repulsion. This prevents aggregation and guarantees steady dispersion.^40^

This negative surface charge is not only important for physical stability but also advantageous in biomedical applications.^64,65^ Additionally, the surface charge plays a role in mediating cellular uptake and biodistribution, particularly in targeted drug delivery and fluorescence-based bioimaging. High negative zeta potential values suggest electrostatic interactions caused by surface charges. These interactions lead to repellent behaviors that hinder aggregation and flocculation. The zeta potential value is a key determinant of nanocomposite suspension behavior during storage. This is critical for several applications, including reducing aggregation during drug delivery.^66^ Therefore, the strongly negative zeta potential of the silica QDs synthesized in this study supports their suitability for biomedical applications.

### 3.4 Optical Properties

Figure 7 conveys the information on silica QDs optical properties described through ultraviolet–visible (UV–vis) absorption and photoluminescence (PL) spectroscopy. Results strengthen their great potential for a variety of applications, particularly in the biomedical field. The UV–vis spectra express a broad optical absorption range of 200–500 nm, with a substantial peak at 282 nm (Figure 7a). The absorption peak at 282 nm represents the intrinsic optical signature of the silica quantum dots, which arise from π–π* transitions in sp^2^-hybridized domains or localized surface states. This is consistent with previous reports where silica-based QDs displayed strong absorption bands in the UV region between 250 and 300 nm due to similar surface state transitions.^67^ Since the synthesized QDs are derived from amorphous silica, no significant shift in absorption edge was observed, aligning with established findings that such structures typically lack sharp excitonic features due to their noncrystalline nature.^68^

**Figure 7:**
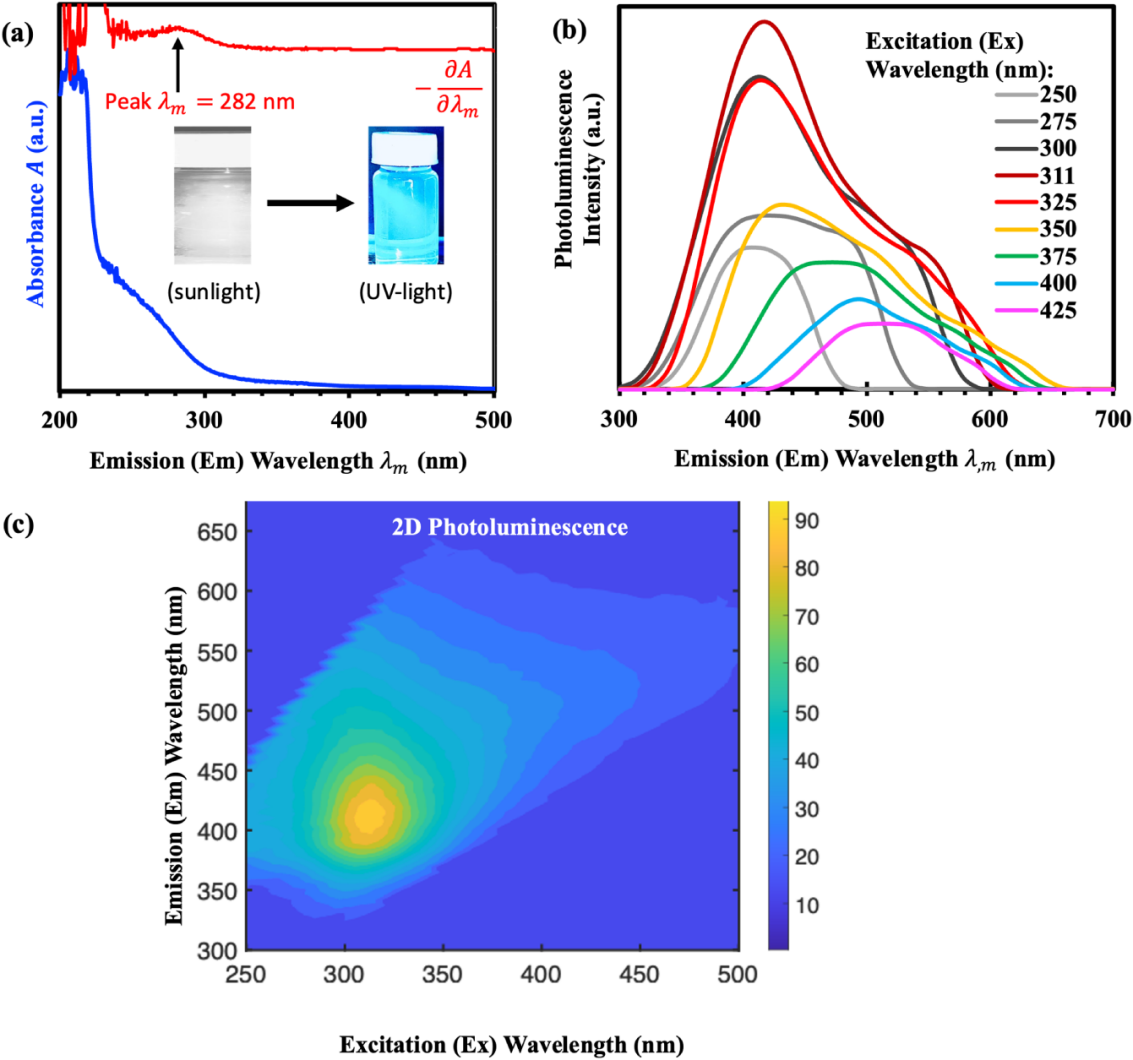
Optical properties measurements: (a) UV–vis absorption of silica QDs solution (blue). Slope analysis of absorbance (red). Inset: photograph of silica QDs solution under sunlight and UV light, (b) photoluminescence spectra of QDs using excitation wavelengths in the range of 250 to 425 nm, and (c) 2D fluorescence map of silica QDs.

Under 365 nm UV excitation, the silica QDs exhibit intense blue luminescence. PL spectra exhibit excitation-dependent behavior with emission wavelengths ranging from 350 to 500 nm as the excitation wavelength is applied at 250 to 425 nm (Figure 7b). The PL emission peak is at 409 nm with the excitation wavelength of 311 nm. This excitation-dependent photoluminescence behavior is a hallmark feature of quantum dot systems. It arises because quantum confinement in nanometer-scale particles leads to discrete energy levels, meaning that smaller particles exhibit larger band gaps and hence higher-energy (blue-shifted) emission. Such size-dependent emission tuning has been reported in silicon nanocrystals and carbon dots, where particle size distributions yield systematic shifts in photoluminescence upon varying excitation energy.^69,70^ Similar behavior about tunable emission from silica QDs in the 400–500 nm range, depending on excitation energy and particle size.^71^ Furthermore, the measured quantum yield (QY) of 20% at 350  nm excitation reflects the efficiency of the light-emitting process, consistent with values typically reported for green-synthesized or surfactant-free silica QDs (commonly 8–20%).^28,31,72^ Figure 7c shows the excitation–emission matrix, indicating a primary excitation peak around 311  nm and reinforcing the multilevel emission nature of the QDs. This property is particularly advantageous for biomedical applications such as multicolor bioimaging.^73^

### 3.5 Cytotoxicity Study

MTT assay was performed to study the cytotoxic effects of silica QDs. Figure 8 reveals a dose-dependent reduction in the viability of both NIH3T3 normal fibroblast and B16F0 melanoma cells. The concentration of silica QDs required to cause a 50% reduction in cell viability, called IC_50_, of NIH3T3 cells is 4532 ppm. IC_50_ values were calculated using a full range of tests at serial concentrations of 15.6, 31.2, 62.5, 125, 250, 500, and 1000 ppm in the MTT assay to assess cell viability. This suggests that silica QDs have low toxicity toward normal cells, thereby, suitable for drug delivery and bioimaging. The IC_50_ for B16F0 melanoma cells was observed at 398.6  ppm, significantly lower than that for NIH3T3 fibroblasts, indicating selective cytotoxicity against melanoma. This differential response asserts the selective toxicity of silica QDs, with greater effectiveness in targeting cancer cells while sparing normal tissue. Relevant studies demonstrate that silica-based nanomaterials frequently induce reactive oxygen species (ROS), which contribute to mitochondrial damage, oxidative stress, and apoptotic cell death, particularly in cancer cells that are more susceptible to oxidative imbalance.^60,74^

**Figure 8:**
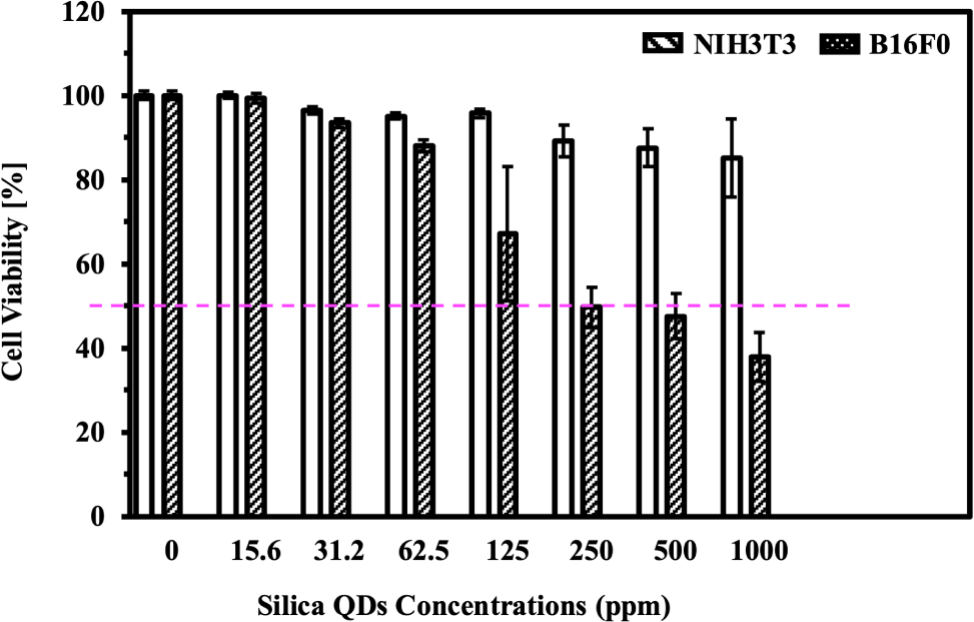
MTT assay cytotoxicity of different concentrations (15.625–1000 μg/mL) of silica QDs measured by MTT assay on NIH3T3 and B16F0 cell lines after 24 h incubation. Note: 1 ppm = 1 μg/mL. Dash pink line indicates a 50% cell viability.

Recent studies have demonstrated that silica QDs induce toxicity in a size-dependent manner, with smaller particles (15 nm) showing significantly higher cytotoxicity than larger ones (50 nm). This toxicity is mediated by oxidative stress, as evidenced by increased reactive oxygen species (ROS) generation.^75^ Another previous study reported that silica QDs induce oxidative stress-mediated cytotoxicity and apoptosis in human epithelial cells (A431 and A549), with A549 cells showing greater sensitivity to silica QDs exposure.^74^ These findings support the hypothesis that the observed selective cytotoxicity in melanoma cells is mediated by ROS mechanisms. This selective vulnerability of cancer cells suggests that the silica QDs could offer a promising approach for therapeutic applications, e.g., used for targeted cancer treatments.^76^

## 4 Conclusions

This paper highlights synthesized silica quantum dots (silica QDs) derived from an abundant geothermal-waste silica, locally extracted from the Geothermal Field at Dieng, Central Java, Indonesia. Therefore, this research expands sustainability aspects in geothermal energy depletion and adheres to green chemistry principles. Associated characterizations, such as X-ray crystallography and spectroscopy, confirm the compositions and properties of typical silica quantum dots. Our studies also justify that the samples exhibit exceptional photoluminescence and biocompatibility, thereby advancing the eco-friendly synthesis and nanomedicine nexus. Summarized observations are as follows:

- Advanced characterization techniques such as field emission scanning electron microscopy (FESEM), high-resolution transmission electron microscopy (HRTEM), and dynamic light scattering (DLS) have proven a uniform distribution at roughly 3 nm and nanoscale precisions, ranging from 2 to 5 nm. These spherical nanosized particles are expected to improve cellular absorption and pose minimal toxicity.
- Zeta potential of −28 mV ensures stable dispersion of silica QDs in suspensions and even biological contexts.
- Tunable optical features (excitation-dependent photoluminescence) and the 20% quantum yield enable the silica QDs to be used for bioapplications such as bioimaging.
- Silica QDs show selective cytotoxicity, with a lower IC_50_ in B16F0 melanoma cells (398.6 ppm) than in NIH3T3 fibroblasts (4532 ppm), indicating potential for targeted cancer therapy due to potentially reactive oxygen species-induced oxidative stress. When functionalized with therapeutic molecules, silica QDs hold significant promise as a targeted diagnostic and therapeutic (theranostic) platform.
- Further studies are needed to investigate surface functionalization with targeting ligands, drug encapsulation efficiency, release kinetics, and cellular uptake mechanisms. Reactive oxygen species generation by silica QDs will also be evaluated using DCFH-DA to better understand their oxidative stress potential and support safe biomedical applications.

## Data Availability

Data for the main figures are available in the NYCU repository: https://doi.org/10.57770/WN8D0Z
